# Characteristics and status of Korean medicine use in whiplash-associated disorder patients

**DOI:** 10.1186/s12906-018-2188-7

**Published:** 2018-04-06

**Authors:** Nohyeon Kim, Byung-Cheul Shin, Joon-Shik Shin, Jinho Lee, Yoon Jae Lee, Me-riong Kim, Eui-Hyoung Hwang, Chan Yung Jung, Diana Ruan, In-Hyuk Ha

**Affiliations:** 1 Jaseng Spine and Joint Research Institute, Jaseng Medical Foundation, 538 Gangnam-daero, Gangnam-gu, Seoul, Republic of Korea; 2 Third Division of Clinical Medicine, School of Korean Medicine, Pusan National University, Yangsan, Republic of Korea; 3 Department of Korean Rehabilitation Medicine, Pusan National University Korean Medicine Hospital, Yangsan, Republic of Korea; 4 College of Korean Medicine, Dongguk University, Gyeongju, Republic of Korea; 5 Columbia University College of Physicians and Surgeons, Columbia University Medical Center, New York, NY USA

**Keywords:** Whiplash injuries, Complementary therapies, Integrative medicine, Acupuncture, Bee venoms, Herbal medicine, Risk factors

## Abstract

**Background:**

Patients are free to choose conventional or Korean medicine treatment under the dual medical system in Korea, and the prevalence of patients who choose Korean medicine treatment for whiplash-associated disorders (WADs) is high. This study analyzed the sociodemographic characteristics and medical service use in this population to provide healthcare providers with basic usage information of complementary and alternative medicine for WAD.

**Methods:**

A total of 8291 outpatients who registered under automobile insurance coverage and visited the main branch of Jaseng Hospital of Korean Medicine from April 1, 2014 to August 10, 2016 were included. Data on sociodemographic characteristics, symptoms, and accident and treatment-related details were collected from electronic medical records. Univariate and multivariate regression analyses were performed to identify baseline factors predictive of total treatment duration.

**Results:**

The most prevalent demographic of patients who chose Korean medicine for WAD treatment was males in their thirties whose initial visit to the hospital was 16.1 ± 94.1 days from the accident. Neck pain accompanied by low back pain (57.0%) was the most common complaint, and for singular pain, neck pain (13.5%) was the most prevalent. Baseline numeric rating scale (NRS) pain levels were generally moderate (4–6) regardless of area. Patients received 7.2 ± 10.2 sessions of treatment for 32.6 ± 55.3 days. The most commonly prescribed treatment modalities in order of highest frequency were acupuncture, cupping, pharmacopuncture, and herbal medicine, which collectively accounted for > 90% of treatments. Acupuncture was administered 29.0 ± 40.8 times, and cupping 14.0 ± 18.7 times as the two highest frequency treatments. In multivariate regression analysis, longer treatment periods were found to be associated with higher NRS, older age, and delayed initial visits at baseline.

**Conclusions:**

This study highlights the characteristics and Korean medicine use of WAD patients. These results are particularly relevant and informative for consideration of personal preferences and effective prioritization in further insurance coverage.

## Background

Traffic accidents (TAs) are among the primary causes of mortality and injury [1], and various musculoskeletal disorders occur as a result of TA-related trauma [2, 3]. Low back pain (LBP) and neck pain are highly prevalent TA-associated symptoms, and the average period for back pain recovery has been reported at 505 days [4]. A total of 1,141,925 cases of TA occurred in 2015 in Korea, resulting in 4621 deaths and 1,809,461 injured people. In addition, the social cost due to property damage, casualties, and communal cost from TA is estimated at 25 million US dollars, which accounts for about 1.8% of South Korea’s 2015 gross domestic product (GDP) [5].

Whiplash can be defined as a transfer of energy to the neck via an acceleration–deceleration mechanism. It usually occurs as a result of rear-end or side-impact automobile collisions. The impact may result in bony or soft-tissue injuries (whiplash-injury), which may in turn lead to a range of clinical manifestations commonly referred to as whiplash-associated disorders (WADs) [6]. Generally, exercise is recommended for mobility and stabilization in treatment of whiplash injuries, and the patient is advised to act as usual rather than engage in pain-avoidance behavior. Non-opioid drugs and nonsteroidal anti-inflammatory drugs (NSAIDs) are commonly prescribed as conventional medications [7]. In several Asian countries such as Korea, various complementary and alternative medicine (CAM) treatments including acupuncture and manipulation are administered for treatment of musculoskeletal disorders [8, 9]. Particularly in Korea, where the medical system employs both traditional Korean and conventional medicine, treatment for WAD encompasses and widely utilizes CAM.

Training of Korean medicine physicians in Korea employs a dual education system where students are required to complete (a) a 6-year undergraduate course consisting of 2 years of pre-medicine and 4 years of medical school adopted by 11 private universities; or (b) a specialized 4-year postgraduate course comprising of 4 years of undergraduate and 4 years of postgraduate courses operated by a national university to graduate and qualify for the national licensing examination for Korean medicine doctors. In the two years of pre-medicine, an introduction to Korean medicine, and courses in liberal arts, medical Chinese, and basic conventional medicine are provided, and in the four years of medical school, various basic and clinical courses are conducted along with clinical training sessions [10].

Many countries have policies addressing WAD-related medical problems, and various research into the occurrence and medical service use of WAD is in progress. In Australia, TA insurance data studies on the status of hospitalization [11] and physical therapy use for WAD have been conducted [12], while a study on mortality rates by age, sex, and TA type has been performed in India [13]. A survey study of general practitioners (GPs), family physicians, and chiropractors was conducted on the treatment of acute whiplash patients in Canada [14], and in Korea, results of treatment use and case reports on WAD patients visiting Korean medicine clinics and hospitals have been published [15], though few studies had large sample sizes. In the ‘2013 National Health Insurance Statistics’ of Korea, Korean medicine was reported to account for about 4.2% of total national health insurance expenditure, while Korean medicine treatment fees amounted to 19.5% of automobile insurance costs in 2014 [16]. The utilization rate of Korean medicine by patients for WAD is higher than the usage rate by patients with national health insurance for non-WAD-related conditions, and this is most likely a result of the fact that various Korean medicine treatments (e.g. herbal medicine, pharmacopuncture, Chuna manual therapy, Do-in (conduction exercise)), which are not under national health insurance coverage, are covered by automobile insurance for WAD patient cases. As automobile insurance covers more otherwise non-payment items within the insurance budget and policy range, and most treatment actions are conducted in the coverage range, it presents a general guide for treatments of maximum effectiveness and satisfaction within limited resources.

However, considering that the insurance company of the assailant bears the medical expenses of the injured party depending on the degree of culpability, WAD treatment follows not only the level of evidence but also TA coverage guidelines set by the Korean Ministry of Land, Infrastructure and Transport and the policy of the Auto Insurance Medical Fee Review Council under the Health Insurance Review and Assessment Service. The current correlation level between clinical practice and insurance and policies is high, and Korean medicine clinical practice guidelines for traffic injuries was initiated in 2016 for standardization of clinical practice, and the guidelines which were published in 2017 are expected to provide a standard for Korean medicine practice in traffic injuries. This current report on Korean medicine treatment of TA injuries was conducted as part of establishing these clinical practice guidelines in an effort to bridge the gap between policy, evidence-based medicine and personal preferences and incorporate them systematically in clinical practice.

Analysis of WAD patients is expected to reflect Korean medicine services preferred by physicians and patients for musculoskeletal disorder treatment and provide valuable data for identifying and understanding patient groups with preference for insurance-supported Korean medicine treatment. This descriptive study examined the sociodemographic characteristics, pain patterns, status of frequency of Korean medicine treatments, and treatment details of WAD patients visiting an integrative medicine hospital for Korean medicine treatment to the aim of providing basic knowledge on this condition to related healthcare providers, researchers, and legislators in Korea, and other countries where complementary and alternative medicine use for WAD is not as prevalent.

## Methods

### Participants

A total of 8291 outpatients who visited the main branch of Jaseng Hospital of Korean Medicine (situated in the Gangnam district of Seoul, Korea) and registered under automobile insurance during the index period from April 1, 2014 to August 10, 2016 were retrospectively analyzed for characteristics and treatment details using electronic medical records. Jaseng Hospital of Korean Medicine is a spine-specializing Korean medicine hospital designated as such by the Korean Ministry of Health and Welfare. Each year, over 900,000 spine and joint patients receive treatment at Jaseng using acupuncture, herbal medicine, and Chuna manual therapy for non-surgical treatment of musculoskeletal disorders. Conventional medicine is utilized for diagnostic assistance and complementary treatment means [17, 18].

### Treatments

Various Korean medicine treatments, such as acupuncture, pharmacopuncture, herbal medicine, cupping, manipulation (Chuna manual therapy, Do-in (conduction exercise)), and traction, are used to treat TA injuries according to the patient’s symptoms under automobile insurance coverage. Pharmacopuncture (also known as herbal acupuncture) is a relatively new acupuncture technique that combines acupuncture and herbal medicine, where herbal extracts are injected into acupoints [19]. In Chuna Manual Therapy, Korean Medicine Doctors (KMDs) apply physical stimuli to the patient’s body to restore balance and function to dysfunction and/or structural problems using medical devices such as Chuna tables [10], and Do-in conduction exercise therapy is a treatment modality that utilizes muscle strengthening exercises and passive joint exercises with individualized controlled breathing to treat abnormal movement and physical dysfunction.

### Collection of data

Upon their first visit to the hospital, patients provided basic sociodemographic information, including their name, sex, date of birth, type of insurance, and mode of hospital entrance using tablet PCs that interwork with the electronic record database. Occupation was categorized into nine types according to the Korean Standard Classification of Occupations (KSCO) [20]. Data on symptoms, such as the date and circumstances of injury related to the symptoms, pain area, severity of pain, smoking and drinking behavior, and comorbidities, were collected by a Korean medicine doctor and also entered into the electronic medical record.

Hospital entrance method was categorized into three types: self-walking when the patient walked in unassisted; assisted walking when walking frames, wheelchairs or outside help was necessitated; and stretcher-dependent when carried in by stretcher-car.

Current pain severity was measured using the numeric rating scale (NRS) (0 representing no pain and 10 the worst pain imaginable) as assessed by the patient and categorized into three stages, 1–3, 4–6, and 7–10, for analysis. Pain regions were divided into three areas: neck pain; LBP; and joint pain (knee or shoulder), and the areas were analyzed both independent of and in combination with comorbid pains. Regarding prior treatment of the current episode, whether the patient had received other treatments, and details of oral analgesic prescription and administration and steroid injections received at other hospitals were recorded.

For radiographic examination, X-rays were referred to the department of radiology, and additional magnetic resonance imaging (MRI), computed tomography (CT), or ultrasonography was taken if deemed necessary. If the patient had prior radiographic imaging results relevant to the current accident, then additional radiographic examinations may have been forgone at the Korean medicine doctor’s discretion.

Treatments are automatically recorded when the Korean medicine doctor responsible for treatment enters prescription codes into the electronic patient records for treatment means. Treatment details were thus determined based on the prescription codes entered into the electronic medical records which were extracted from the electronic database and comprehensively reviewed by the researchers, and the treatment administration details were analyzed by a designated statistician.

### Statistical analysis

The basic characteristics, initial treatment timepoint, total treatment duration, radiological diagnosis, and Korean medicine treatment details of WAD patients who visited a Korean medicine hospital were analyzed. The treatment period was set from the first hospital visit to the last treatment date recorded in the hospital electronic system. When the patient had suffered two or more TAs within the index period and had been required to register again, the two events were analyzed separately. Demographic characteristics, past medical history, pain, and treatment service use are presented as descriptive statistics. Continuous variables were calculated by mean and standard deviation, and nominal variables by frequency and percentage (%). For analysis of predictive factors at baseline that affected total treatment period, univariate/multivariate regression analyses were performed. Statistical testing was performed with a significance level of 0.05. All statistics were analyzed using SAS 9.4 (SAS Institute Inc., Cary, North Carolina, USA) and STATA 14.0 (StataCorp, College Station, Texas, USA).

### Ethics, consent and permissions

This study was approved by the Institutional Review Board of Jaseng Hospital of Korean Medicine (approval number: JASENG 2017–05-006; approval date: June 5th, 2017) and adhered to all relevant research ethics. Written informed consent was obtained from patients to access and use medical records for academic means.

## Results

The total number of TA patients who visited the hospital for WAD treatment within the index period was 8291. The average age was 36.0 ± 10.9 years, and 55.7% were male. First hospital visits were made an average 16.1 ± 94.1 days after the accident. Of these patients, 32.9% visited the hospital within a day, 38.7% within 2–7 days, 11.9% within 8–14 days, and 16.5% after more than 14 days, which shows that most of the patients visited the hospital within a week of sustaining their injury.

In prior treatments for WAD-related neck pain and LBP episodes, 552 patients with neck pain and 428 patients with LBP took analgesics an average of 7.4 ± 6.5 and 7.9 ± 6.8 times, respectively. Fifty patients with neck pain and 71 patients with LBP took steroids, which displays a relatively higher use by LBP patients, but the average number of times of steroids taken was comparable for neck pain and LBP at 2.2 ± 1.8 and 2.1 ± 2.1 times, respectively. In comparisons of previous interventions and operations, only non-surgical interventions were analyzed, as no neck pain patients or LBP patients had received surgery for their current pain (Table 1).

**Table 1:** Sociodemographic characteristics, medical comorbidities, and prior treatment for current symptoms

	*N*	% (including individuals with missing data)
Age (years), mean ± SD	8291	36.0 ± 10.9
< 20	200	2.4
20 ≤ < 30	1953	23.6
30 ≤ < 40	3730	45.0
40 ≤ < 50	1432	17.3
50 ≤ < 60	649	7.8
≥ 60	327	3.9
Sex		
Male	4617	55.7
Female	3674	44.3
First visit from onset (days), mean ± SD	7878	16.1 ± 94.1
0 ≤ ≤1	2588	31.2
2 ≤ ≤7	3050	36.8
8 ≤ ≤14	941	11.3
> 14	1299	15.7
Missing data	413	5.0
Occupation		
Professionals	1844	22.2
Office workers	1751	21.1
Sales or service workers	1428	17.2
Administrative managers	820	9.9
Technicians and engineering-related workers	102	1.2
Equipment, machine operating and assembling workers	60	0.7
Manual laborers	47	0.6
Armed forces	27	0.3
Skilled agriculture, forestry and fishery workers	8	0.1
Inoccupation (including housewives and students)	2106	25.4
Missing data	98	1.2
Smoking^a^		
Current smoker	2618	31.6
Former smoker	214	2.6
Non-smoker	5248	63.3
Missing data	211	2.5
Drinking^b^		
Yes	3820	46.1
No	4457	53.8
Missing data	14	0.2
Hospital entrance method		
Self-walking	7979	96.2
Assisted walking	45	0.5
Stretcher-car	3	0.0
Missing data	264	3.2
Comorbidity^c^		
Hypertension	335	4.0
Liver disease	249	3.0
Diabetes	138	1.7
Other musculoskeletal disease	103	1.2
Cardiovascular disease	88	1.1
Gastrointestinal disease	39	0.5
Pulmonary disease	30	0.4
Depression	4	0.0
Other	719	8.7
Prior treatment for present illness^d^		
Analgesics intake for neck pain, mean ± SD	552	7.4 ± 6.5
Steroids intake for neck pain, mean ± SD	50	2.2 ± 1.8
Intervention for neck pain, mean ± SD	11	1.3 ± 0.6
Analgesics intake for LBP, mean ± SD	428	7.9 ± 6.8
Steroids intake for LBP, mean ± SD	71	2.1 ± 2.1
Intervention for LBP, mean ± SD	49	1.3 ± 1.2

*LBP* low back pain

^a^Current smoker: individuals who currently smoke and have smoked one or more packs of cigarettes within the past year

Former smoker: individuals who previously smoked one or more packs of cigarettes in their lifetime and have smoked less than a pack of cigarettes within the past year

Non-smoker: individuals who have smoked less than a pack of cigarettes in their lifetime

^b^Drinking, yes: individuals who have drunk one bottle of soju or beer or more within the past month

^c^Previously diagnosed comorbidities as self-reported by patient

^d^Treatments prescribed for current pain episode before presenting at current hospital

The distribution of pain was divided into three groups for analysis: neck pain, LBP, and joint pain (shoulder or knee). Each appeared independently in 13.5%, 7.0%, and 1.5% of patients, respectively, and neck pain combined with LBP was reported by 57.0% of patients, which indicates a high prevalence of accompanying spinal pain. With regard to concurrent spinal and joint pain, 1.6% of patients had LBP and joint pain, 1.8% neck pain and joint pain, and 5.0% all three (neck pain, LBP, and joint pain) together. The severity of pain was analyzed in four groups (LBP; neck pain; shoulder pain; and knee pain) by baseline NRS, which were reported on average as 5.1 ± 1.4 for LBP, 5.1 ± 1.4 for neck pain, 5.4 ± 1.4 for shoulder pain, and 5.0 ± 1.5 for knee pain. These results show that baseline NRS scores were all in the 4–6 category, regardless of the pain area (Table 2).

**Table 2:** Distribution of pain area and severity

		*N*	% (excluding individuals with missing data)
Pain distribution	Single pain area		
	Neck pain with or without radiating pain	1547	13.5
	Neck pain with radiating pain^a^	420	5.1
	LBP with or without radiating pain	939	7.0
	LBP with radiating pain^a^	353	4.3
	Joint pain (shoulder or knee)	146	1.8
	Shoulder pain	47	0.6
	Knee pain	78	0.9
	Multiple pain areas		
	Neck pain with LBP	4723	57.0
	LBP with joint pain	135	1.6
	Neck pain with joint pain	150	1.8
	Neck pain and LBP with joint pain	412	5.0
Severity of current pain^b^	Baseline NRS of LBP in all LBP patients, mean ± SD	6213	5.1 ± 1.4
	1 ≤ ≤3	647	10.4
	4 ≤ ≤6	4849	78.1
	7 ≤ ≤10	717	11.5
	Baseline NRS of LBP in LBP with radiating pain patients, mean ± SD	2010	5.0 ± 1.5
	1 ≤ ≤3	331	16.5
	4 ≤ ≤6	1430	71.1
	7 ≤ ≤10	249	12.4
	Baseline NRS of neck pain in all neck pain patients, mean ± SD	6843	5.1 ± 1.4
	1 ≤ ≤3	685	10.0
	4 ≤ ≤6	5384	78.7
	7 ≤ ≤10	774	11.3
	Baseline NRS of neck pain in neck pain with radiating pain patients, mean ± SD	2069	4.9 ± 1.5
	1 ≤ ≤3	400	19.3
	4 ≤ ≤6	1444	69.8
	7 ≤ ≤10	225	10.9
	Baseline NRS of shoulder pain, mean ± SD	328	5.4 ± 1.40
	1 ≤ ≤3	34	10.37
	4 ≤ ≤6	230	70.12
	7 ≤ ≤10	64	19.51
	Baseline NRS of knee pain, mean ± SD	623	5.0 ± 1.5
	1 ≤ ≤3	93	14.9
	4 ≤ ≤6	446	71.6
	7 ≤ ≤10	84	13.5

^a^Subgroup of category immediately above

^b^Evaluated in all patients with relevant pain regardless of number of comorbid pains

*LBP* low back pain, *NRS* numeric rating scale

The total treatment period was less than a week for 3304 patients (33.9%), 2–4 weeks for 2436 patients (29.4%), 5–12 weeks for 1678 patients (20.3%), 13–26 weeks for 625 patients (7.5%), and more than 26 weeks for 244 patients (2.9%). Patients received 7.2 ± 10.2 sessions of treatment over an average of 32.6 ± 55.3 days. In order of decreasing frequency, the types of Korean medicine treatment administered were acupuncture, cupping, pharmacopuncture, and herbal medicine, which collectively accounted for more than 90% of treatment prescriptions. On average, patients treated using acupuncture or cupping, the two most commonly used treatments, received acupuncture 29.0 ± 40.8 times, and cupping 14.0 ± 18.7 times.

Of the patients subjected to conventional diagnostic imaging, 7470 received X-rays, and an average of 2.4 ± 1.1 exams were performed on these patients. CT examinations were performed an average of 1.0 ± 0.1 times in 162 patients, and MRI was performed 1.1 ± 0.3 times in 1070 patients. X-rays, CTs, and MRIs were conducted at 0.2 ± 4.5, 2.8 ± 7.8, and 20.0 ± 27.5 days after the patient’s first visit to the hospital, respectively (Table 3).

**Table 3:** Treatment duration, diagnostic imaging and Korean medicine service use of traffic accident patients

	*N*	%	Period, mean ± SD	Number of treatment sessions, mean ± SD
Treatment duration (weeks), mean ± SD	8287		32.6 ± 55.3	7.2 ± 10.2
0 ≤ ≤1	3304	39.9	1.9 ± 2.5	1.7 ± 0.9
2 ≤ ≤4	2436	29.4	16.7 ± 5.9	5.1 ± 2.5
5 ≤ ≤12	1678	20.3	48.8 ± 15.2	11.2 ± 6.8
13 ≤ ≤26	625	7.5	120.2 ± 26.9	21.7 ± 13.6
> 26	244	2.9	270.4 ± 82.5	39.1 ± 23.4
Korean medicine treatment				
Acupuncture	7991	96.4		29.0 ± 40.8
Cupping	7769	93.7		14.0 ± 18.7
Pharmacopuncture	7659	92.4		6.3 ± 7.2
Herbal medicine	7532	90.9		4.8 ± 5.8
Do-in (conduction exercise)	5478	66.1		5.6 ± 6.8
Chuna manual therapy	4210	50.8		5.8 ± 7.3
Traction	297	3.6		6.1 ± 7.7
Diagnostic imaging				
X-ray	7470	90.1	0.2 ± 4.5^a^	2.4 ± 1.1
MRI	1070	12.9	20.0 ± 27.5^a^	1.1 ± 0.3
CT	162	2.0	2.8 ± 7.8^a^	1.0 ± 0.1
Ultrasound	3	0.0	44.0 ± 34.0^a^	1.0 + 0.0

^a^Period up to examination: period between first hospital visit and examination date; Recorded as 0 when examined on first day of hospital visit

*MRI* Magnetic Resonance Imaging, *CT* Computed Tomography

Univariate and multivariate regressions were performed on factors such as sex, age, occupation, smoking and drinking behavior, height and weight, comorbidity, baseline pain levels, and first visit to the hospital since the accident to identify baseline factors predictive of the total treatment period. Higher baseline NRS, greater patient age, and delayed first visits to the hospital correlated with longer treatment periods (Table 4).

**Table 4:** Analysis of baseline factors potentially predictive of total treatment duration

Factors	Univariate regression		Multivariate regression	
	Standardized beta	*p*	Standardized beta	*p*
Sex (female) (Ref. male)	0.077425	< 0.001	0.0381054	0.117
Age	0.1317144	< 0.001	0.0828506	< 0.001
Baseline NRS of LBP	0.1126834	< 0.001	0.0801922	< 0.001
Baseline NRS of neck pain	0.0952296	< 0.001	0.0290658	0.095
First visit from onset	0.0691402	< 0.001	0.0996183	< 0.001
Comorbidity (yes)	0.0760425	< 0.001	0.0290279	0.053
Height	−0.048538	< 0.001	0.0017629	0.941
Weight	−0.0415678	< 0.001	− 0.0101693	0.657
Occupation (professional) (Ref. inoccupation)	0.0096079	0.47	0.0203401	0.246
Occupation (office worker) (Ref. inoccupation)	−0.0088438	0.504	0.0097252	0.582
Occupation (sales or service worker) (Ref. inoccupation)	−0.0555756	< 0.001	−0.0243107	0.161
Occupation (other) (Ref. inoccupation)	−0.00714	0.572	−0.0051363	0.762
Smoking (current smoker) (Ref. non-smoker)	−0.1084881	< 0.001	−0.0643908	< 0.001
Smoking (former smoker) (Ref. non-smoker)	−0.012401	0.266	0.0012622	0.931
Drinking (yes)	−0.0331203	0.003	0.0065889	0.66

*NRS* numeric rating scale, *LBP* low back pain

**Fig. 1:**
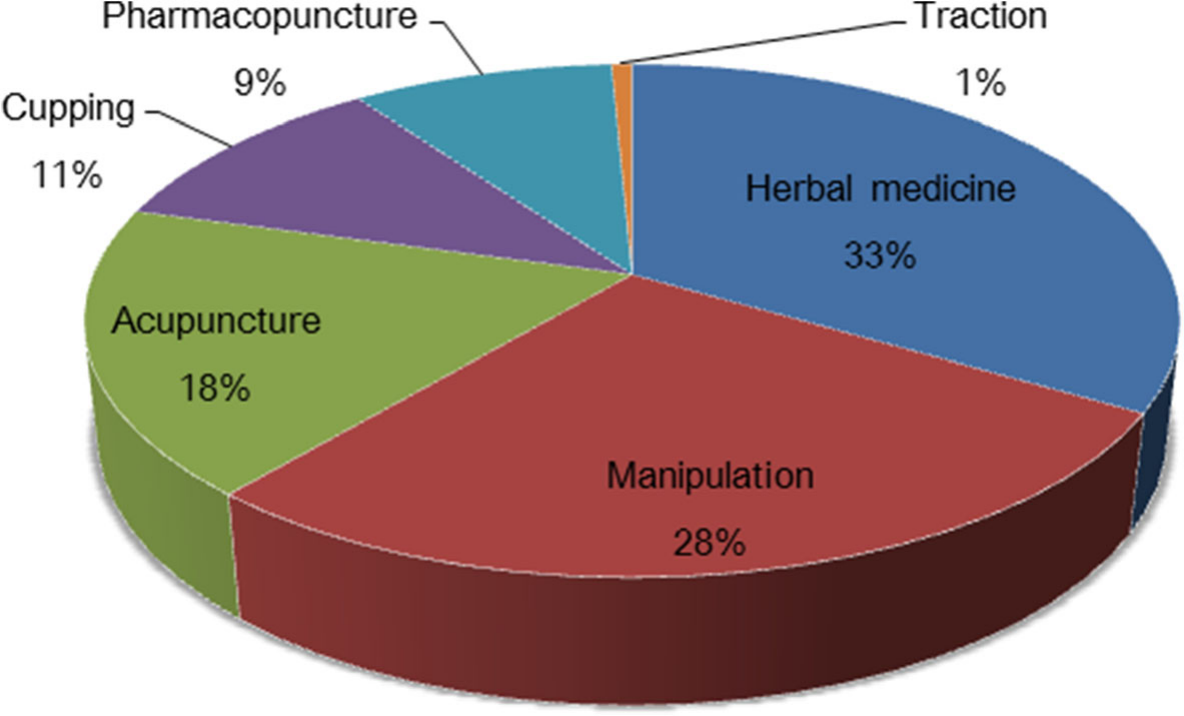
Total cost of Korean medicine treatments

The percentages of costs claimed for each intervention during the total treatment period were, in descending order, herbal medicine (33%), manipulation (28%), acupuncture (18%), cupping (11%), pharmacopuncture (9%), and traction (1%) (Fig. 1).

## Discussion

This study examined the demographic characteristics, distribution and severity of symptoms, treatment period and frequency, and details of high frequency diagnostic testing and treatments in WAD patients visiting an integrative medicine hospital for Korean medicine treatment. The greatest number of WAD patients was male patients in their 30s who were office workers, and only 30% of WAD patients continued to receive treatment after four weeks and at decreasing frequencies. Most patients were examined using X-ray imaging, and some underwent additional MRIs, CTs, or ultrasound. Frequently used Korean medicine treatments for WAD patients consisted of acupuncture, pharmacopuncture, cupping, and herbal medicine. In addition, the total duration of treatment tended to be longer in patients of older age, with higher baseline NRS of LBP levels, and with delayed first visits to the hospital after the accident.

Most TA patients who visited the hospital were in their 30s, so considering the fact that most patients who use Korean medicine are in their 40s, the population of traffic accident patients using Korean medicine is relatively young [21]. While Ahn et al. analyzed medical service use patterns and high frequency treatments in Korean national insurance claims data of LBP patients and determined that patients with musculoskeletal pain tended to be female [22], this study found that the percentage of males with musculoskeletal pain was higher in WAD patients. It could be speculated that this is associated with the occupational and environmental differences between males and females of working age (15–64 year old males *n* = 14,027,000; females *n* = 10,364,000), and the fact that the main hospital branch is located in a highly populated urban district with high work traffic intensity. This could also be related to the overall occupational and population distribution of Korea and/or Seoul [23].

Patients first visited the hospital an average of 16.1 ± 94.1 days after the accident, and this average seems high when compared to the median (3 days; 1st and 3rd quartiles: 1 day, 9 day, respectively). In some cases, patients visited the hospital for the first time after considerable time had lapsed, which subsequently increased the mean period between the onset of injury and their first visit to the hospital. Meanwhile, most patients visited the hospital within ten days, which appears to be associated with the acute manifestation of WAD symptoms following the accident. Complaints of single area symptoms appeared as neck pain, LBP, and joint pain, which indicates that neck pain was the main symptom among WAD patients. Furthermore, over half reported neck pain combined with LBP, and this combination was also the most prevalent complaint reported. An additional 10.2% of patients reported joint pain, such as shoulder or knee pain.

The reason X-ray examinations were conducted most frequently and in the shortest time was to verify the magnitude of the injury (i.e. to rule out fractures). Comparing MRI and CT, CT was performed in addition to X-ray examinations within a relatively short period - as early as 2.8 ± 7.8 days after the first hospital visit - which may be attributed to the need to further diagnose fractures and potential causes of bleeding in higher resolution. MRI was conducted within 20.0 ± 27.5 days to diagnose residual neurological symptoms and damage to soft tissue. To rate WADs, X-ray examination is required for differentiation of fractures, and previous studies report the evaluation of muscle and tendon injuries using MRI [24, 25]. However, although X-ray and MRI are frequently used and various guidelines recommend early imaging for older adults, study results have shown that early imaging for back pain is not helpful for the treatment and prognosis of older adults [26]. This suggests the need to factor in disease and patient characteristics, and appropriate timeframe and frequency when considering additional imaging studies.

Generally, treatment lasted for less than a month (69.3%) or for 3 months (cumulative percentage 89.6%), but cases that required longer periods of treatment also reached 10.4%. Investigation of the association between baseline risk factors and long-term treatment revealed that current smoking, higher baseline NRS of LBP, older age, and a longer period between the first hospital visit and pain onset were correlated with longer treatment periods. The individual appearances of LBP and radiating pain were not as common as neck pain, but the baseline NRS of LBP exerted a large influence on prognosis. To shorten the treatment period and increase treatment effectiveness, patient education on the importance of early visits to the hospital after TA and lifestyle management appears necessary.

The analysis of the total cost of Korean medicine treatment shows that while acupuncture and cupping are the most frequently used (acupuncture 96.4%, and cupping 93.7%, respectively), their cost percentages compared to the total cost are only 18% and 11%, and herbal medicine and Do-in (conduction exercise) costs are high. The differences among these cost ratios can be attributed to the high per session costs of herbal medicine and manipulation sessions. In prescription of herbal medicine, preparation expenses including the price of herbal ingredients, and decocting and shipping charges are included in the total cost, and prescription orders are usually filled in 7 to 10 day units. Further, the legal definition of manipulation requires Korean medicine doctors to conduct manipulation treatment for 10 min or longer, which results in higher per session costs compared to other more prevalent procedures such as acupuncture.

### Strengths and limitations

Some strengths of this study include that it covers the greatest number of patients compared to previous studies of Korean medicine treatment in TA patients, to the best of our knowledge. This study also uses hospital medical records as opposed to insurance databases or physician surveys to capture patient sociodemographic and pain characteristics, as well as the utilization status of high frequency Korean medicine treatments and treatment details of patients visiting an integrative medicine hospital to receive Korean medicine treatment. With the exception of a few interventions such as acupuncture and cupping, many commonly used Korean medicine treatments are not covered by Korean national health insurance. Therefore, the previous literature on the use of Korean medicine for general musculoskeletal diseases reflects the substantial out-of-pocket costs of Korean medicine treatment and its limited insurance coverage availability. However, in TA patients the distribution of the use of various treatments in analysis of Korean medicine is less influenced by cost because most Korean medicine treatment modalities for TA patients are covered by automobile insurance. Rather, the distribution is more reflective of physician and patient preferences, which holds significance in prioritizing treatment options in Korean medicine and/or CAM to improve and strengthen national health insurance coverage. For example, pharmacopuncture and herbal medicine were used to similar extents as acupuncture, which supports their clinical relevance in treating musculoskeletal diseases. Reduction of medical expenses through shorter treatment duration is also an important issue in treatment of TA patients, and this study provides basic data on various patient characteristics and their implications.

This study also has several limitations. This is a retrospective study of electronic medical records, and some data may have been erroneously entered or omitted owing to computer or human error. Missing data were subsequently excluded from analysis. In addition, as recorded hospital visits were used to calculate patient treatment periods, patient visit information as recorded in the electronic system may differ from the actual date of treatment initiation and conclusion. Moreover, clinical improvement scales such as pain intensity, functional scale and quality of life (QoL), satisfaction, and other treatment effect measures were not included as this study mainly pertained to administrative records. Lastly, although automobile insurance covers a wider range of treatment than national health insurance, treatment administration is still partially limited, and the results should therefore be interpreted with caution. For example, Do-in (conduction exercise) and Chuna manual therapy are required to be prescribed separately to qualify for automobile insurance reimbursement and cannot be prescribed simultaneously, and coverage of herbal medicine is curtailed beyond 20 days.

## Conclusions

Through the analysis of medical records of WAD patients who visited an integrative medicine hospital for Korean medicine treatment, this study comprehensively examined patient characteristics and distribution, pain patterns, and treatment period and details of WAD patients receiving Korean medicine treatment. The results of this study are expected to contribute basic knowledge for related healthcare service providers, researchers, and legislators to effectively prioritize items for insurance coverage and in establishing clinical practice guidelines for traffic injuries.

## Abbreviations

CAM: Complementary and alternative medicine
CT: Computed tomography
GDP: Gross domestic product
GNI: Gross national income
GP: General practitioner
KSCO: Korean Standard Classification of Occupations
LBP: Low back pain
MRI: Magnetic resonance imaging
NRS: Numeric rating scale
NSAID: Nonsteroidal anti-inflammatory drug
TA: Traffic accident
WAD: Whiplash-associated disorder
